# LipL32 Is a Subsurface Lipoprotein of *Leptospira interrogans*: Presentation of New Data and Reevaluation of Previous Studies

**DOI:** 10.1371/journal.pone.0051025

**Published:** 2013-01-08

**Authors:** Marija Pinne, David A. Haake

**Affiliations:** 1 Research Service, Veterans Affairs Greater Los Angeles Healthcare System, Los Angeles, California, United States of America; 2 Division of Infectious Diseases, Veterans Affairs Greater Los Angeles Healthcare System, Los Angeles, California, United States of America; 3 Department of Medicine, David Geffen School of Medicine, University of California Los Angeles, Los Angeles, California, United States of America; 4 Department of Urology, David Geffen School of Medicine, University of California Los Angeles, Los Angeles, California, United States of America; 5 Department of Microbiology, Immunology & Molecular Genetics, University of California Los Angeles, Los Angeles, California, United States of America; Royal Tropical Institute, The Netherlands

## Abstract

The agents of leptospirosis, a zoonosis with worldwide distribution, are pathogenic spirochetes belonging to the genus *Leptospira*. The leptospiral life cycle involves transmission via fresh water and colonization of the renal tubules of their reservoir hosts. Infection of accidental hosts, including humans, may result in life-threatening sequelae. Bacterial outer membrane proteins (OMPs), particularly those with surface-exposed regions, play crucial roles in pathogen virulence mechanisms and adaptation to environmental conditions, including those found in the mammalian host. Therefore, elucidation and characterization of the surface-exposed OMPs of *Leptospira* spp. is of great interest in the leptospirosis field. A thorough, multi-pronged approach for assessing surface exposure of leptospiral OMPs is essential. Herein, we present evidence for a sub-surface location for most or all of the major leptospiral lipoprotein, LipL32, based on surface immunofluorescence utilizing three different types of antibodies and four different permeabilization methods, as well as surface proteolysis of intact and lysed leptospires. We reevaluate prior evidence presented in support of LipL32 surface-exposure and present a novel perspective on a protein whose location has been misleading researchers, due in large part to its extraordinary abundance in leptospiral cells.

## Introduction

Leptospirosis, a zoonosis caused by pathogenic *Leptospira* spp. transmitted from rodents and other reservoir hosts to humans via contaminated water, has a significant public health impact in tropical and sub-tropical regions [1]–[5]. Leptospirosis also has significant adverse effects on the agricultural industry, causing abortions, infertility, and death in livestock [6], [7]. After being shed in the urine of a reservoir host animal, leptospires may persist for months in freshwater or wet soil, providing opportunities for contact with abraded skin or mucous membranes of a new host. In an accidental host, the resulting infection is potentially fatal, and is frequently characterized by jaundice, renal failure, and/or pulmonary hemorrhage [1], [4], [8]. As a result, there is great interest in identification of surface-exposed outer membrane proteins (OMPs) with the capacity to serve as vaccine antigens.

The two major types of leptospiral OMPs, outer membrane lipoproteins and transmembrane OMPs, differ significantly in their structure and how they are associated with the outer membrane. Lipoproteins become associated with membranes via a hydrophobic interaction between the N-terminal acyl moieties and the phospholipids of the lipid bilayer [9], [10]. Lipoproteins can be localized to one or more of four cellular compartments: the periplasmic leaflet of the inner membrane, the periplasmic or outer leaflets of the outer membrane, or the extracellular space [9], [10]. Notably, the bioinformatic algorithm, SpLip, is suitable for prediction of spirochetal protein lipidation but does not address the cellular destination of lipoproteins [11].

The goal of this study was to apply a comprehensive experimental strategy, together with re-evaluation of previously published findings, to assess the localization of the major leptospiral lipoprotein, LipL32. Previously, leptospiral OMP identification relied on subcellular fractionation methods, including Triton X-114 detergent extraction-phase partitioning and the isolation of OM vesicles [12]–[15]. These approaches work well for the differentiation of OM from inner membrane lipoproteins [12], [16], [17]. However, these methods are not applicable for assessment of protein surface-exposure. Recently, we developed a comprehensive surface-localization strategy involving several complementary methods to identify and characterize proteins located on the leptospiral surface. The surface proteolysis method and our extensive immunofluorescence assays allowed us to determine that LipL32 is largely or exclusively a sub-surface protein. This finding forced us to re-examine previously published data [12], [17]–[19] in support of LipL32 surface-exposure. We believe that these earlier data are actually more consistent with a sub-surface location for LipL32 and therefore, in agreement with the findings presented here. We propose that the extreme abundance of LipL32 [20] has led to artifactual results that were misinterpreted when damaged organisms were present in surface-exposure assays. Our findings do not compromise the localization of LipL32 as an outer-membrane protein, as it is most likely tethered to the inner leaflet of the lipid bilayer. It is anticipated that the data presented here will provide new perspectives on this protein and facilitate studies to elucidate the role(s) of LipL32 in *Leptospira* biology.

## Materials and Methods

### Ethics statement

This study was conducted according to principles expressed in the Declaration of Helsinki. Informed written consent was obtained from participants and the study was approved by the Institutional Review Board A of the Research and Development Committee, VA Greater Los Angeles Healthcare System (PCC #2012 - 050702).

Co-Author David A. Haake has a patent on leptospiral protein LipL32. This does not alter our adherence to all PLoS ONE policies on sharing data and materials.

### Bacterial strains and growth conditions

*Leptospira interrogans* serovar Copenhageni strain Fiocruz L1-130 was isolated from a patient during a leptospirosis outbreak in Salvador, Brazil [5]. Leptospires were cultivated at 30°C in Probumin™ Vaccine Grade Solution (84-066-5, Millipore, Billerica, MA) diluted five-fold into autoclaved distilled water [21]. Competent *E. coli* NEB 5-α (New England Biolabs, Ipswich, MA), and BLR(DE3)pLysS (Novagen, Madison, WI) were used for cloning and expression, respectively. *E. coli* were grown in Luria-Bertani (LB) broth or on agar plates with 50 µg/ml carbenicillin, 12.5 µg/ml tetracycline, 34 µg/ml chloramphenicol, 40 µg/ml kanamycin or 40 µg/mlspectinomycin (Sigma-Aldrich, St. Louis, MO) when appropriate.

### Gel electrophoresis, antibodies and immunoblotting

Protein samples were boiled for 5 min in Novex NuPage sample buffer (Life Technologies, Carlsbad, CA) in the presence of 2.5% β-mercapthoethanol and separated through Bis-Tris 4–12% polyacrylamide gradient NuPage gels using the Novex XCell Sure Lock electrophoresis cell (Life Technologies).

The polyclonal rabbit sera specific for the following proteins are described elsewhere: FlaA2 [18], OmpL37, OmpL47, OmpL54 [21], LipL31 [12], OmpL1 [22], LipL41 [23], and LipL32 [17]. LipL32 monoclonal antibody 1D9 [24], [25] was a kind gift from Dr. José Antonio Guimarães Aleixo (Universidade Federal De Pelotas, Pelotas, Brazil). Patient sera from leptospirosis outbreaks in 1996 and 1997 in Salvador, Brazil, were kindly provided by Dr. Albert I. Ko (Yale University School of Public Health, New Haven, CT). Leptospirosis patient serum samples were prepared by pooling convalescent sera from 23 individuals with laboratory-confirmed leptospirosis. Normal human serum (ImmunoPure) was obtained from Thermo Scientific (Rockford, IL).

For immunoblotting, proteins were transferred to a polyvinylidene difluoride (PVDF) Immobilon-P membrane (Millipore, Billerica, MA) and probed with rabbit polyclonal antisera or LipL32 antibodies affinity-purified from leptospirosis patient sera. Bound antibodies were detected using horseradish peroxidase (HRP)-conjugated anti-rabbit IgG (GE Lifesciences, Buckinghamshire, England), or anti-human IgG (Sigma-Aldrich, St. Louis, MO), respectively. Immunoblots were visualized by enhanced chemiluminescence reagents according to the manufacturer's instructions (Thermo Scientific).

### Affinity purification of LipL32 antibodies from leptospirosis patient sera

Two mg of recombinant LipL32 [17] were coupled to the AminoLink Plus column according to manufacturer's instructions (Thermo Scientific). Convalescent sera from 23 individuals with laboratory-confirmed leptospirosis were pooled and 800 µl was added to 3.7 ml of 10 mM phosphate buffered saline, pH 7.4 (PBS) followed by filtration through 0.45 µm filter. Two ml of filtered sera was added to the affinity column and mixed by rotation for 1 h at room temperature. One ml of PBS added to the column, the flow-through (FT) fraction was collected and the rest of filtered sera (2.2 ml) was added to the column repeating the process as described above. The column was washed four times with 2 ml of PBS and LipL32-specific antibodies were recovered by addition of IgG elution buffer (Thermo Scientific) to the affinity column.

### Membrane fractionation

For membrane affinity experiments, total membranes were isolated as described previously [26]. Briefly, 5×10^9^ leptospiral cells were washed twice with PBS, containing 5 mM MgCl_2_ and resuspended in 0.9 ml of lysis buffer (10 mM TrisHCl, pH 8.0, 5 mM EDTA, 0.5% protease inhibitor cocktail, Sigma-Aldrich) containing 1 mg/ml of lysozyme. The suspension was incubated for 5 min at 4°C and subjected to three cycles of freezing (−80°C) and thawing (room temperature) with vigorous vortexing. Then DNase I (Sigma-Aldrich) was added to a final concentration of 5 µg/ml and the cell suspension was incubated on ice for 20 min. Membranes were recovered by centrifugation at 16,000× g for 15 min at 4°C and resuspended in 0.5 ml of lysis buffer (without lysozyme). A 100 µl aliquot of the membrane suspension was mixed with 100 µl of either 0.2 M Na_2_CO_3_, 3.2 M urea, 1.2 M NaCl, or lysis buffer and incubated for 15 min at 4°C. The samples were pelleted at 16,000× g for 15 min at 4°C and the supernatants were precipitated with acetone. Each membrane pellet and its supernatant precipitate were resuspended in 50 µl of Novex NuPage sample buffer (Invitrogen, Carlsbad, CA).

### Cell surface proteolysis of intact *Leptospira* cells

*L. interrogans* Fiocruz L1-130 was grown to the density of 2–6×10^8^ cells/ml and harvested by low-speed centrifugation at 2,000× *g* for 7 min at room temperature. Assessment of surface exposure of leptospiral proteins on intact cells was performed by Proteinase K treatment as previously described [21]. To evaluate the capability of Proteinase K to digest LipL32, cell lysates were prepared by solubilizing leptospires in 50 mM Tris-HCL (pH 8.0), 100 mM NaCl, 2 mM ethylenediaminetetraacetic acid (EDTA), 0.2% sodium dodecyl sulfate (SDS) and boiled for 5 min. Proteinase K was added directly to the cell lysates and performed as previously described [21] with an exception that the centrifugation and washing steps were omitted.

### Surface immuno-fluorescence (IFA) assay

*L. interrogans* cultures at densities of 2×10^8^ to 5×10^8^ cells/ml were harvested by low-speed centrifugation at 2,000× *g* for 7 min at room temperature and surface exposure of proteins was done by IFA as previously described [21], [27]. As controls to demonstrate antibody recognition of subsurface proteins, additional outer-membrane permeabilization methods other than methanol fixation/permeabilization were employed to eliminate the possibility that antibodies for LipL32 recognize methanol-denaturated form of protein more efficiently. For permeabilization by PBS, cells were resuspended in PBS, vortexed for 30 sec and centrifuged at 14,000× *g* for 5 min at room temperature, repeating this procedure one more time before adding a 1-ml suspension of 5×10^8^ spirochetes to each well of Lab-Tek Two-Well Chamber Slides (Nalge Nunc, Naperville, IL) and incubated at 30°C for 80 min to adhere cells. For permeabilization by EDTA, cells were resuspended in PBS+ 2 mM EDTA and to Lab-Tek Two-Well Chamber Slides. For permeabilization by shear force, cells were resuspended in PBS and pressed through a 28 5/8 gauge needle with a syringe repeating the process four times before adding suspension Two-Well Chamber Slides. For these permeabilization methods, bacteria were fixed to the glass slides by incubation for 40 min at 30°C in 2% paraformaldehyde in PBS-5 mM MgCl_2_.

Antibodies were diluted in blocking buffer (Difco *Leptospira* Enrichment EMJH, BD, Sparks, MD) as follows: rabbit serum recognizing LipL32 1∶800, affinity-purified antibodies from leptospirosis patient serum recognizing LipL32 1∶300, monoclonal antibodies for LipL32 1∶800, rabbit sera recognizing OmpL54 1∶50, and FlaA2 1∶600. Normal human serum was diluted 1∶300. Alexa Fluor 488-labeled goat anti-rabbit IgG, goat anti-mouse IgG or goat anti-human IgG (Invitrogen/Molecular Probes, Eugene, OR) diluted 1∶2000 and fluorescent nucleic acid stain, 4′6-diamidino-2-phenyl-indole dihydrochloride (DAPI) (Invitrogen/Molecular Probes) diluted to a final concentration of 0.25 µg/ml in blocking buffer were used to detect antibody binding and the presence of spirochetes, respectively.

## Results

### Surface proteolysis does not degrade LipL32

**Figure 1:**
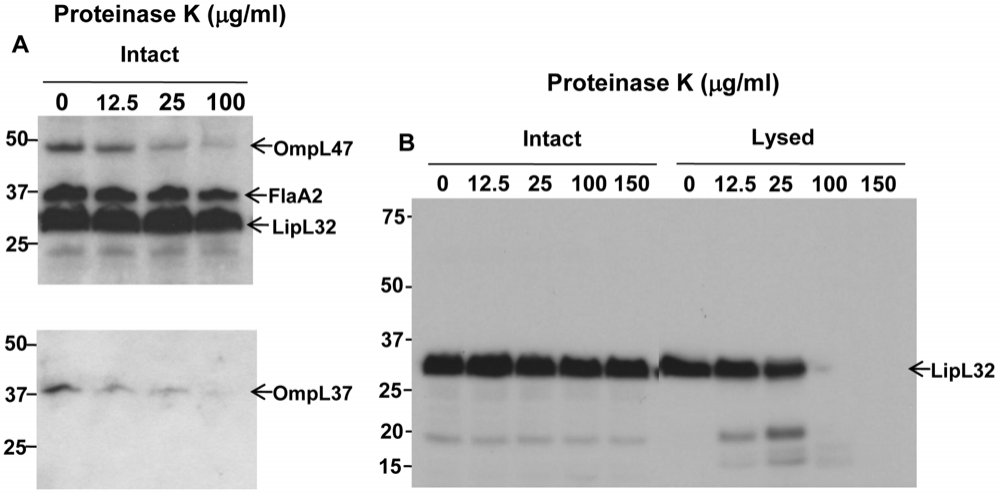
Surface localization of *L. interrogans* serovar Copenhageni strain Fiocruz L1-130 proteins by protease K treatment. (A) Whole intact spirochetes were incubated with different concentrations of Proteinase K. 1×10^8^ of leptospires per lane were separated by gel electrophoresis (Bis-Tris 4–12% NuPage gel, Novex), transferred to a PVDF membrane, and probed with polyclonal rabbit antisera against: LipL32, OmpL47, OmpL37, FlaA2 and LipL31. (B) Whole intact leptospires and cells lysed with 50 mM Tris-HCL (pH 8.0), 100 mM NaCl, 2 mM EDTA, 0.2% SDS and boiling for 5 min were treated as above and probed with rabbit serum recognizing LipL32. The data is representation of four experiments performed separately. The identities of individual proteins are indicated on the right, and the positions of molecular mass standard (in kilodaltons) are indicated on the left.

Surface proteolysis experiments involving incubation of intact leptospires with Proteinase K were performed to assess surface exposure of leptospiral proteins. Based on the assumption that LipL32 is a surface-exposed lipoprotein, previous surface proteolysis in our laboratory had included LipL32 as positive control. Surprisingly, LipL32 was not digested by Proteinase K at concentrations capable of digesting surface-exposed proteins OmpL47 and OmpL37 (Fig. 1A). To eliminate the possibility that LipL32 is intrinsically resistant to Proteinase K cleavage, intact and lysed leptospiral cells were subjected to proteolysis showing efficient cleavage of LipL32 in lysed cells but not in intact cells, suggesting a subsurface location for LipL32 (Fig. 1B).

### LipL32 is not detected on the surface of intact leptospires by IFA using various antibodies

**Figure 2:**
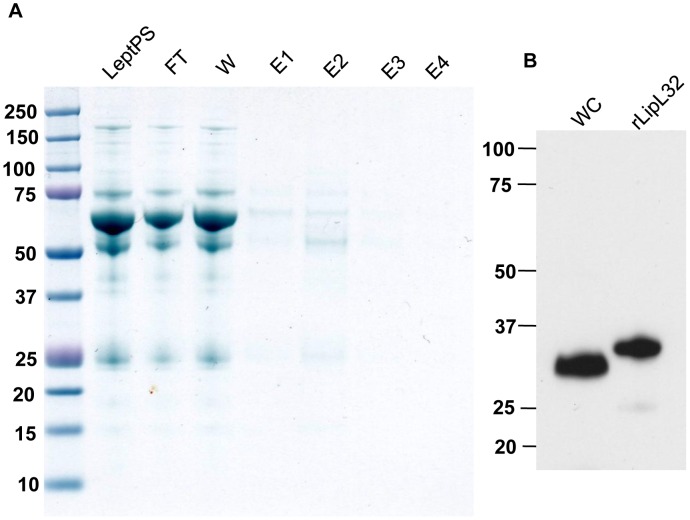
Purification and specificity of LipL32 antibodies from leptospirosis patient sera. (A) Affinity purification of LipL32-specific antibodies. Recombinant LipL32 [17] was coupled to an AminoLink Plus column. Pooled convalescent sera from 23 individuals with laboratory-confirmed leptospirosis was added to the LipL32-affinity column. The chromatography products were analyzed by gel electrophoresis (Bis-Tris 4–12% NuPage gel, Novex), and Coomassie G250 staining. Abbreviations: LeptoPS, leptospirosis patient sera (pooled); FT, flow-through fraction; W, fraction after washing with PBS; E1-E4, eluted IgG fractions. (B) Extract of 1×10^8^ leptospires (lane WC) or 0.5 µg of recombinant LipL32 (lane rLipL32) were separated by gel electrophoresis, blotted onto PVDF membrane, and probed with affinity purified LipL32 IgG fraction E2 (1∶200).

**Figure 3:**
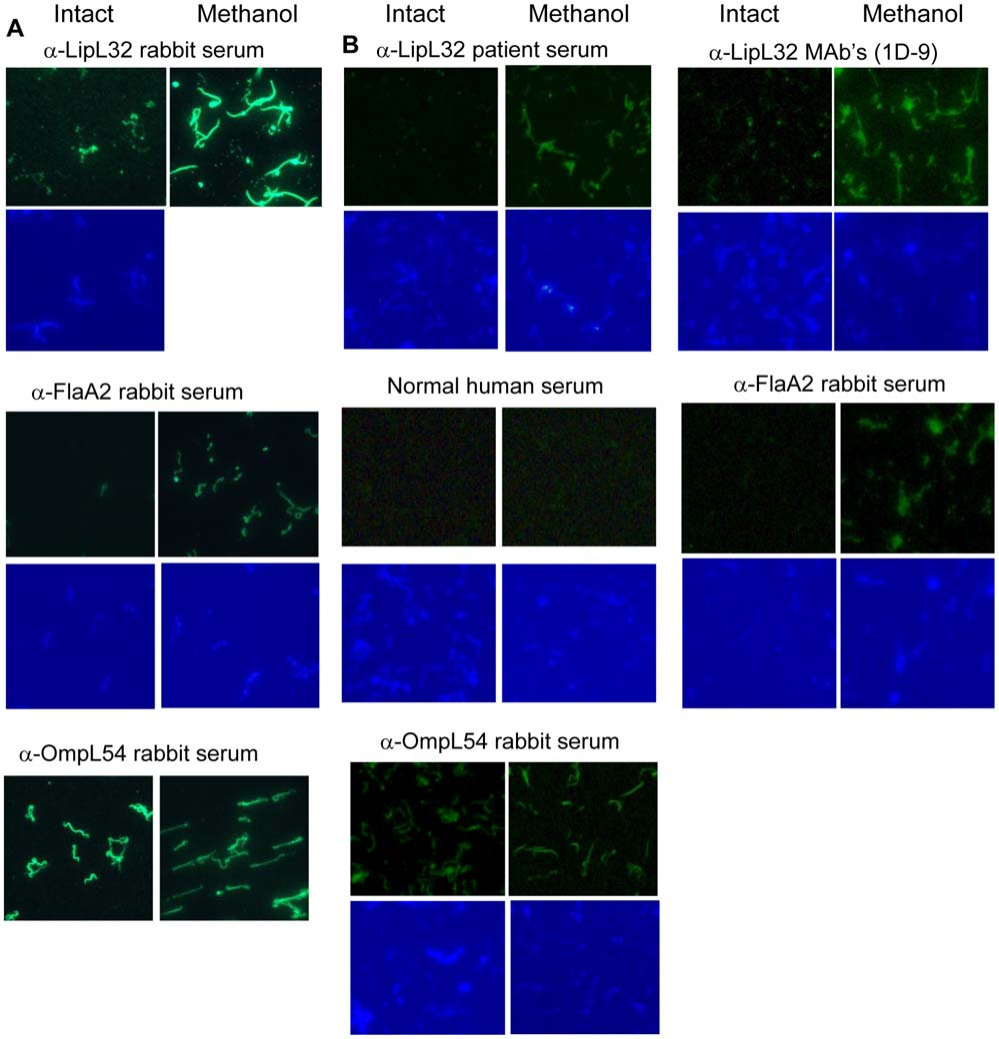
Localization of LipL32 by surface immunofluorescence assay (IFA). Intact or membrane-permeabilized spirochetes were probed with immune sera. Binding of rabbit antibodies to leptospires was detected with Alexa Fluor 488 conjugated goat anti-rabbit IgG fragments. Binding of LipL32 monoclonal antibodies was detected with Alexa Fluor 488 conjugated goat anti-mouse IgG fragments. Binding of LipL32 antibodies purified from leptospirosis patient sera were detected with Alexa Fluor 488 conjugated goat anti-human IgG fragments. A DAPI counterstain was used to demonstrate the presence of spirochetes. The data is representation of four (A) or three (B) experiments performed separately. The identities of individual proteins recognized by the particular antibody reagent are indicated on the top of each column.

**Figure 4:**
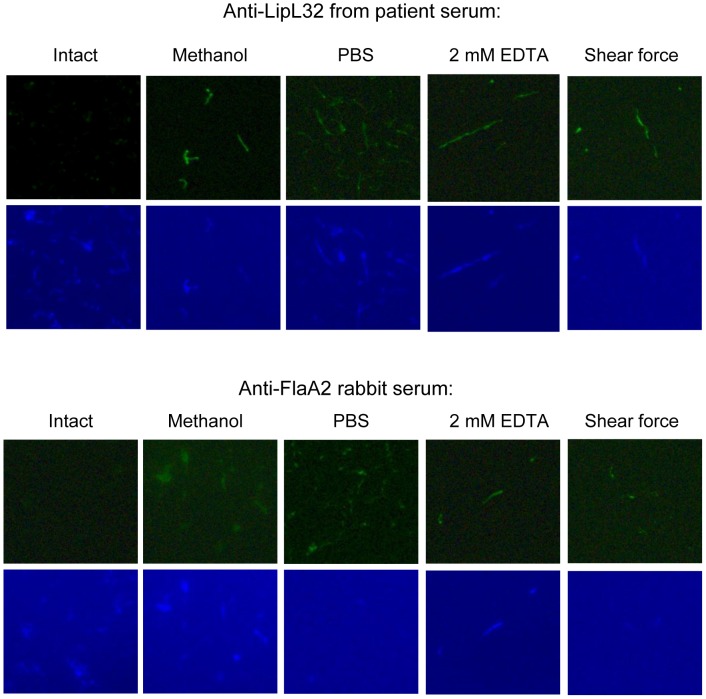
Confirmation of subsurface locale of LipL32 by surface IFA and various outer-membrane permeabilization methods. Intact spirochetes or cells disrupted by methanol, vortexing and high-speed centrifugation, 2 mM EDTA or shear force were probed with affinity purified LipL32 antibodies from leptospirosis patient sera or FlaA2 rabbit serum as a control. The data is representation of three experiments performed separately. Binding of antibodies to leptospires were detected either with Alexa Fluor 488 conjugated goat anti-human IgG fragments (for LipL32) or Alexa Fluor 488 conjugated goat anti-rabbit IgG fragments (for FlaA2). A DAPI counterstain was used to demonstrate the presence of spirochetes. The identities of individual proteins recognized by the particular antibody reagent are indicated on the top of each column.

A variety of antibody reagents recognizing LipL32 were employed to reduce the risk of false negative results resulting from a failure to recognize surface-exposed epitopes. In addition to anti-LipL32 rabbit serum [10] and monoclonal antibodies for LipL32 [24], [25] raised against whole protein, LipL32-specific antibodies from human clinical leptospirosis sera were obtained by affinity purification (Fig. 2). Chromatography was performed by applying pooled convalescent sera from leptospirosis patients on a recombinant LipL32-affinity column and eluting specific IgGs as fractions E1-E4 (Fig. 2A). Pure and specific antibodies recognizing both native and recombinant LipL32 were obtained in elution fraction 2, E2 (Fig. 2A and B). Surface immunofluorescence assays utilizing these three different types of antibodies revealed that LipL32 was readily recognized by anti-LipL32 rabbit serum, monoclonal antibodies or affinity-purified antibodies from leptospirosis patient sera only after the OM was permeabilized by methanol (Fig. 3). Antibodies against sub-surface FlaA2 were included to assess the integrity of the leptospiral OM, showing that sub-surface proteins are exposed only after OM permeabilization (Fig. 3). Positive control experiments were performed with antibodies recognizing OmpL54, a known surface-exposed protein (Fig. 3). Normal human serum was used as a negative control to eliminate the possibility that the signal obtained by affinity purified LipL32 IgGs were due to non-specific binding by cross-reactive antibody species in human serum (Fig. 3). These data clearly demonstrate that LipL32 is not detected on the surface of intact *L. interrogans* by IFA (Fig. 3). To further strengthen this conclusion, mechanical and chemical OM disruption methods, including vortexing and high-speed centrifugation in PBS, chelation with 2 mM EDTA and shear force by passing organisms through a narrow needle, were tested to exclude the possibility that the antibodies selectively recognized methanol-denatured LipL32. Immunofluorescence experiments with affinity purified anti-LipL32 IgGs revealed that LipL32 is recognized only after disruption of the OM without a substantial difference between the permeabilization methods applied (Fig. 4). Experiments with anti-FlaA2 serum was utilized to assess permeabilization efficiency, demonstrating that while methanol appears to be the most effective permeabilization agent, the three other methods also resulted in OM disruption (Fig. 4).

### LipL32 is associated with the leptospiral membrane

**Figure 5:**
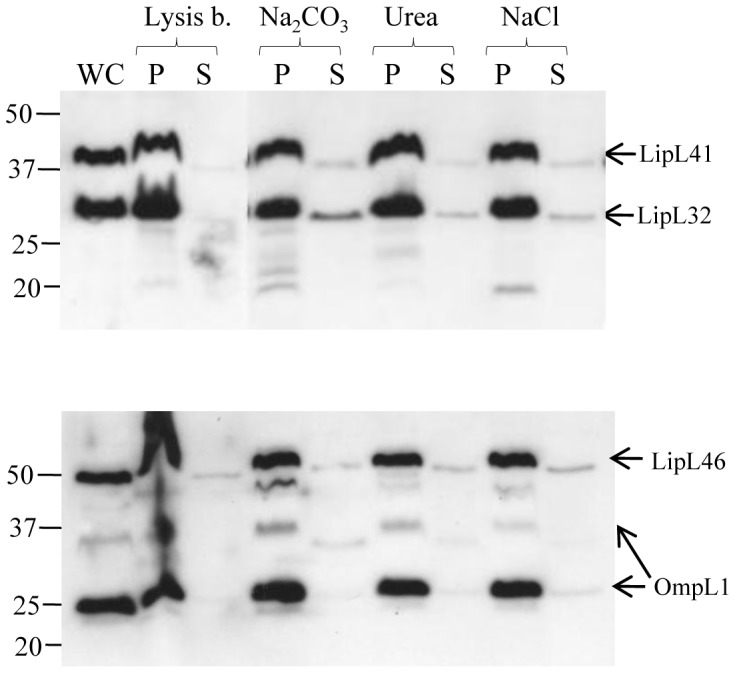
Membrane affinity analysis of LipL32, LipL41, LipL46 and OmpL1. The membrane fraction of *L. interrogans* was treated with lysis buffer as a control or 0.1 M Na_2_CO_3_ (pH 11), 1.6 M urea, or 0.6 M NaCl for 15 min at 4°C. Samples were pelleted by centrifugation to separate the membrane pellet (P) and soluble supernatant (S), followed by gel electrophoresis (Bis-Tris 4–12% NuPage gel, Novex), and immunoblotting with specific antisera. Lane WC contained the whole cell unfractionated lysate of *L. interrogans*. The location of individual proteins are indicated on the right, and the positions of molecular mass standard (in kilodaltons) are indicated on the left.

Membrane affinity analysis was performed to determine whether LipL32 is associated with the lipid bilayer. Treatment of bacterial cells with lysozyme and several freeze-thaw cycles, followed by centrifugation separates proteins into soluble (cytoplasmic and periplasmic) and pellet (total membrane) fractions [28]. The membrane fraction was treated with high pH (0.1 M Na_2_CO_3_), high salt (0.6 M NaCl), or urea (1.6 M), to release peripheral membrane proteins not anchored in the lipid bilayer [21], [26], [29]–[31]. Immunoblot analysis of the soluble (supernatants) and insoluble (pelleted) membrane fractions revealed that the bulk of LipL32 remained associated with the membrane fraction after all treatments (Fig. 5). Integral outer membrane protein OmpL1, and two OM-lipoproteins; LipL46, and LipL41 were included as positive controls and could not be released from the membrane by any treatment (Fig. 5;[26], [30]). As a positive control for release from the membrane, the effect of treatments on the peripheral membrane protein, P31_LipL45_, also known as Qlp42 [32] was also assessed. Substantial release from the membrane by urea and Na_2_CO_3_ was observed (data not shown), as previously described [21], [30].

## Discussion

LipL32 is the most abundant protein in pathogenic *Leptospira* [17], [20] and arguably the most widely studied protein in leptospirosis research [17], [24], [25], [33]–[36]. The lipoprotein nature of LipL32 and its presence in outer-membrane fraction was previously reported [17]. Previous studies have also reported that LipL32 is exposed on the leptospiral surface [18]. Here we report surface-proteolysis and immunofluorescence assays performed to re-evaluate the localization of LipL32. We show that LipL32 on intact leptospires is not cleaved by Proteinase K, whereas the enzyme digests the protein efficiently in lysed cells (Fig. 1). When performed with both positive and negative controls, as we have done here, this result clearly suggests that the bulk of LipL32 is not surface exposed. To further evaluate LipL32 surface exposure, we conducted IFA studies utilizing three different types of LipL32 antibodies. In each case, LipL32 was recognized only after the outer membranes were permeabilized with methanol (Fig. 3). To eliminate the possibility that LipL32 antibodies are recognizing only methanol-denaturated protein, the IFA was performed using different OM-permeabilization methods, showing that regardless of which method was used to perturb the OM, LipL32-specific antibodies recognize the protein only in disrupted cells (Fig. 4). While our surface localization data clearly indicate that LipL32 is not exposed on the leptospiral surface, LipL32 was confirmed as an integral membrane protein (Fig. 5). Although the membrane affinity methods do not discriminate between outer and inner membrane proteins, LipL32 has been previously localized to the outer membrane by Triton X-114 fractionation [17] and membrane vesicle fractionation [12]. LipL32 is completely solubilized by Triton X-114 fractionation, but a significant amount of LipL32 found in protoplasmic cylinder fraction by membrane vesicle fractionation [12], most likely due incomplete separation of outer membrane from inner membrane vesicles rather than inner membrane localization.

**Figure 6:**
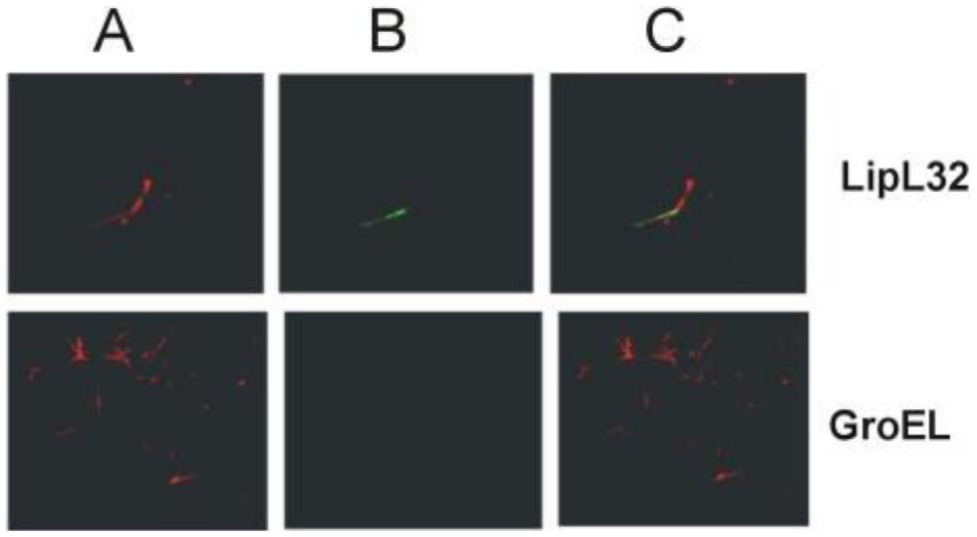
Reused from: PLoS One. 2011; 6(7): e21962. Confocal microscopy was performed with live *L. interrogans* using antisera specific for LIC10258, LIC12880, LIC12238, LipL32 (surface-exposed lipoprotein) and GroEL (protoplasmic cylinder marker). FITC-conjugated secondary antibodies were used to detect the surface-bound antibodies (B). Leptospires were identified by propidium iodide (A) staining of the DNA. Co-localization is shown in the merged images (C).

Our results showing a subsurface location for LipL32 appear to contradict previous studies. This prompted us to reexamine the evidence for LipL32 surface localization presented in previous studies. Immunoelectron microscopy of intact leptospires was presented as evidence for LipL32 surface-exposure [18]. However, given the abundance of LipL32, significantly more immunogold staining should have occurred than what was observed. For example, immunoelectron microscopy of *Borrelia burgdorferi* using OspC antibodies results in dense staining of the surface of the organism with gold particles [37]. When surface immunofluorescence was performed with rabbit serum recognizing LipL32 [18], much weaker and irregular antibody labeling was obtained in intact cells when compared to permeabilized cells. One possible explanation is that this labeling resulted from damaged organisms presented in that particular microscopic field. When LipL32 was used as a positive control in previously published IFA experiments [19], [38], LipL32 surface-exposure was inconclusive as only one of two cells was labeled by antibodies in one study (Fig. 6) [19], while only one cell per microscopic field was shown in the other study [38]. LipL32 monoclonal antibodies [24], [25] have also been utilized in IFA, however the interpretation of the data is impossible given the lack of controls for the integrity of the outer membrane [24]. In fact, when we assessed LipL32 surface exposure using these same monoclonal antibodies, we found that the antibodies recognized the protein only after the OM have been disrupted (Fig. 3). Out of concern about the ability of antibody reagents to recognize native vs. denatured LipL32 epitopes, we also performed immunofluorescence assays with IgG's purified from human clinical leptospirosis sera. These results support the conclusion that most, if not all, LipL32 is not exposed on the surface of intact leptospiral cells.

Surface biotinylation is a widely accepted method for identifying surface proteins and has been employed to demonstrate that LipL32 is exposed on leptospiral surface [18]. However, the published results show that LipL32 is surface-biotinylated in much smaller amounts than would have been expected [18] and that cytoplasmic GroEL and periplasmic FlaB1 were labeled as well, indicating the presence of damaged cells in the biotinylation experiment. Another possibility is that only certain isoforms of LipL32 may reside on the leptospiral surface as previously suggested [18]. Further evidence that LipL32 may not be surface-exposed comes from whole cell ELISA data presented by Cullen and coworkers [18]. Even though LipL32 is three times more abundant that LipL41 [20], surface labeling by LipL32 antiserum is considerably weaker than that of LipL41, particularly when optimal number of cells (7×10^8^ per well) with varying antisera dilutions are utilized [18]. Importantly, when compared to the whole cell ELISA, sonicated leptospires were about 10 times more reactive [18], indicating that LipL32 is either exclusively subsurface or that only a fraction of the cellular LipL32 protein population is accessible to antibody. The steric hindrance by LPS has been given as an explanation for more efficient antibody binding to LipL32 when cells are lysed by Cullen and coauthors [18]. Our IFA results utilizing various disruption methods that do not lyse the cells completely nor strip the LPS from the outer membranes, still showed much stronger signal in disrupted cells compared to intact leptospires (Fig. 4). Moreover, LPS steric hindrance would be expected to apply to antibody or Proteinase K based detection assays for other characterized surface-exposed OMPs, which does not appear to be the case [21], [38]–[40]. While further studies are necessary to obtain a clearer picture of the localization and function of LipL32, our results indicate that this protein is not a good choice as a positive control in protein surface-localization studies.

There have been reports on extracellular-matrix (ECM) component binding abilities of LipL32 [33], [34]. However, ECM binding is not a particularly strong argument for surface exposure as LipL32 binding avidity is relatively weak, antibodies for LipL32 did not inhibit leptospiral binding [34] and a *lipL32* transposon mutant is equally adherent to ECM [36].

Taken together, the surface proteolysis and immunofluorescence data presented here, as well as our reassessment of previous studies, strongly point towards the conclusion that LipL32 is largely, if not exclusively, a subsurface membrane lipoprotein. The abundance of LipL32 represents a major investment of energy and resources by leptospiral cells. This investment and the high level of LipL32 amino acid sequence conservation [41] suggests an important functional role in pathogenic *Leptospira* cells. Although immunization by LipL32 did not elicit protection in hamsters [35] and LipL32 is not required for either acute or chronic infection by *L. interrogans* [36], it should not be assumed that this protein is unimportant for leptospires *in vivo*. In fact, LipL32 is expressed at high levels during infection based on antibody reactivity with LipL32 in 94% of convalescent sera from leptospirosis patients [42] and detection by immunohistochemistry in the kidney [17] and blood [43] of infected animals. Some studies [44], [45] have reported that LipL32 can elicit strong immune response or even act as partially protective antigen when presented to immune system by certain delivery systems, such as Cholera toxin B subunit [44] or *Mycobacterium bovis* BCG [45]. However, generation of anti-LipL32 antibodies is not evidence for surface exposure as it is widely recognized that an immune response to immunogenic cytoplasmic proteins, such as GroEL and DnaK, frequently occurs during infection, including during leptospirosis [46]. It is possible that LipL32 function may be affected by posttranslational modification events. The carboxy-terminus of LipL32 undergoes proteolytic cleavage both *in vitro* [16] and *in vivo* [47]. Moreover, LipL32 is both phosphorylated and methylated [48], which warrants further studies on this intriguing protein. Despite the availability of detailed crystal structure data [49], [50], the primary function(s) of LipL32 remain largely unknown. Nevertheless, we hope that our reassessment of this protein's subcellular location will assist investigators in formulating and testing novel hypotheses regarding the role of LipL32 in pathogenic *Leptospira* species.
